# Enamel matrix derivative (EMD) enhances the osteogenic differentiation of bone marrow mesenchymal stem cells (BMSCs)

**DOI:** 10.1080/21655979.2021.1971504

**Published:** 2021-09-30

**Authors:** Lu Cheng, Ying Li, Qian Xia, MaoHua Meng, ZhaoYang Ye, ZhengLong Tang, HongChao Feng, Xin Chen, HeLin Chen, Xiao Zeng, Yi Luo, Qiang Dong

**Affiliations:** a Department of Prosthodontics, School of Stomatology, Guizhou Medical University, Guiyang, Guizhou Province, 550004, People’s Republic of China; b Department of Prosthodontics, Guiyang Hospital of Stomatology, Guiyang, Gsuizhou Province, 550002, People’s Republic of China; c Clinical Research Center, the Affiliated Hospital of Guizhou Medical University, Guiyang, Guizhou Province, 550004, People’s Republic of China; d Department of Prosthodonticsand Oral Implantology, Stomatological hospital of Guizhou Medical University, Guiyang, Guizhou Province, 550004, People’s Republic of China; e Department of Oral and Maxillofacial Surgery, Guiyang Hospital of Stomatology, Guiyang, Guizhou Province, 550002, People’s Republic of China

**Keywords:** Enamel matrix derivative, bone marrow mesenchymal stem cells, Wnt/β-catenin signaling

## Abstract

To investigate the EMD’s capacity in BMSCs osteogenic differentiation. In vivo and in vitro, BMSCs were treated with EMD, scanning electron microscopy, and Alizarin Red staining were used to detect the changes in the osteogenic ability of BMSCs, and the proliferation ability of BMSCs was evaluated by CCK8. In addition, by adding xav939, a typical inhibitor of Wnt/β-catenin signaling pathway, the regulatory function of Wnt/β-catenin signaling was clarified. The results showed that EMD promote cell proliferation and 25 μg/ml EMD had the most significant effect. Cells inducing osteogenesis for 2 and 3 even 4 weeks, the cell staining is deeper in EMD treated group than that of the control (P < 0.05) by alizarin Red staining, suggesting more mineralization of BMSCs. In vivo implanting the titanium plate wrapped with 25 μg/ml EMD treated-BMSC film into nude mice for 8 weeks, more nodules were formed on the surface of the titanium plate than that the control (P < 0.05). HE showed that there is a little blue-violet immature bone-like tissue block. Besides, the expression of RUNX Family Transcription Factor 2 (Runx2), Osterix, Osteocalcin (OCN), collagen I (COLI), alkaline phosphatase (ALP) and β-catenin were inhibited in xav939 group (P < 0.05); Inversely, all were activated in EMD group (P < 0.05). In conclusion, EMD promoted the proliferation and osteogenic differentiation of BMSCs. EMD’s function on BMSCs might be associated with the Wnt/β-catenin signaling pathway.

## Graphical abstract

**Figure:**
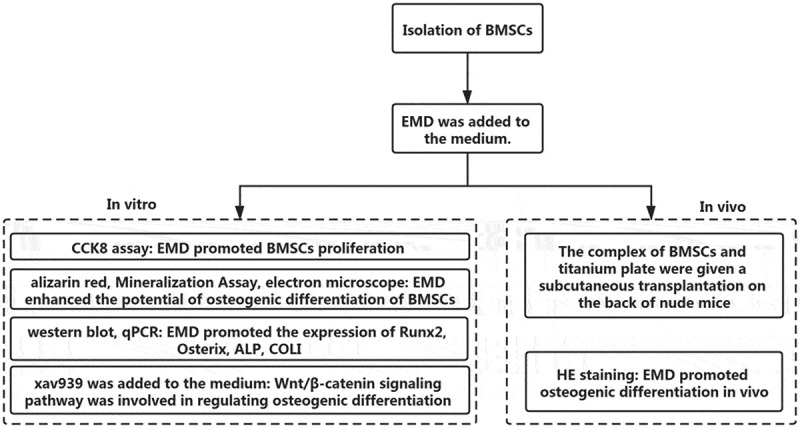

## 1 Background

At present, due to the advantages of no damage to natural abutments and high masticatory efficiency, dental implant restoration has been accepted by more and more patients with dentition defects [1]. However, due to trauma, inflammation, and other factors, teeth loss are often accompanied by varying degrees of alveolar bone defect, and the amount of alveolar bone around the titanium implant is often the key to determine the long-term successful use of the titanium implant [2]. Therefore, bone regeneration therapy is often needed in dental implantation to ensure that there is enough healthy bone tissue around the titanium implant [3]. At present, autografts are deemed as the gold standard for bone grafts because of their excellent histocompatibility and nonimmunogenic properties. Nevertheless, autologous bone transplantation needs to open up a new bone donor area, the available bone quantity is limited, and there may be various complications [4]. For serious bone defects, multiple bone grafting operations are required but the results of bone grafting are uncertain [5]. Mesenchymal stem cells (MSCs) are a cluster of multipotent, clonogenic cells dwelling in many organs’ stroma, for instance, bone marrow, adipose tissue, muscle, and liver, in which they play an important role. Given that their pleiotropic function and broad tissue distribution, MSCs are regarded as the relevant targets to enhance tissue regeneration [6].

EMD is made from the enamel matrix proteins of minor porcine teeth [7]. EMD’s major component is amelogenin, a family of hydrophobic proteins, making up of more than 90% of the total protein content [8]; the others are the enamelins, such as proline-rich enamelin, sheathelin, and tuftelin [7,9]. However, EMD’s efficiency on the osteogenesis of stem cells has been controversial. On the one hand, some studies argued that EMD results in a reduction in osteoblasts differentiation [8]. Although EMD is widely utilized in periodontal treatments and the new cementum is often observed [8], relevant report on alveolar bone formation remains scant [10,11]. On the other hand, EMD was also reported to stimulate cellular proliferation and the mineralization of both pre-osteoblasts and osteoblasts [12–14], resulting in an inhibition in the myogenic and lipogenic differentiation of pluripotent mesenchymal cells and leading to the induction of their differentiation into osteoblasts and chondrocytes [13,15].

EMD has albeit been widely studied for its functions on cell proliferation and differentiation in vitro and its clinical application in periodontitis, EMD’s effect on BMSCs in vitro remains vacant [16,17]. It is believed that the canonical Wnt signaling pathway is player in the regulation of MSCs osteogenic differentiation [18]. Furthermore, Wnt signaling pathway also leads to β-catenin’s accumulation within the cytoplasm and finally contributes to the translocation into the nucleus as a transcriptional auxiliary activator. Canonical Wnt signaling activation facilitates bone formation through multiple routes, ranging the promotion of the mesenchymal stem cells’ differentiation into mature osteoblasts to the enhancement in the proliferation and the mineralization of osteoblasts [19,20]. It has been revealed the regulation of canonical Wnt signaling in osteogenesis-related cytokines expressions [21–23]. However, EMD’s correlation with the BMSCs’ osteogenic differentiation of canonical Wnt pathway is yet to be explored.

We hypothesized that EMD could promote the proliferation and osteogenic differentiation of BMSCs through regulating Wnt/β-catenin signaling pathway. Therefore, this study was aimed to investigate EMD’s effect on BMSCs and to analyze its mechanism. This would provide experimental basis for an exploration onto effective stem cell therapy for bone regeneration in the field of oral implants.

## 2. Materials and methods

### 2.1 Isolation and culture of BMSCs

The isolation and the culture of SPF grade SD rat bone marrow mesenchymal stem cells in vitro in a way of whole-bone marrow adherent method **[**24**]**. First of all, the cells were cultured in complete medium containing DMEM (Gibco, Carlsbad, CA, USA) supplemented with 10% fetal bovine serum (Biological Industries, Israel), 100 U/mL penicillin (Sigma–Aldrich, USA). Then, the plates were incubated in a humidified atmosphere at 37^◦^C in 5% CO_2_. After the first 24 h half-change of media, the media was changed every 3 days. By the time, the cells fused to 80%–90%, digestion and passage were conducted at 1:3, and the 3rd to 6th generation was used for follow-up experiments.

### 2.2 Flow cytometric analysis on cell surface markers

The surface markers of bone marrow mesenchymal stem cells were determined by flow cytometry. After BMSCs were digested with trypsin, PBS (Biological Industries, Israel) was resuspended and counted. 1*10^6^ cells/tube were added to the flow up sample tubes to be boiled into single-cell suspension. CD45, CD29, CD90, CD11b primary antibody (BioLegend, San Diego, CA, USA) were added to the tubes, respectively. The negative control group was incubated with the same amount of staining buffer for 30 minutes at 4℃. Finally, the samples were detected by flow cytometry [25].

### 2.3 Osteogenic and adipogenic differentiation

BMSCs were inoculated in 6-well dishes with 5 × 10^5^ cells/well and cultured to 90% fusion in complete medium and then induced by osteogenic induction medium (complete medium supplemented with 100 nM dexamethasone, 10 mM β-Glycerophosphate disodium salt hydrate, 50 μM 2-Phospho-L-ascorbic acid trisodium salt (Sigma-Aldrich, USA)). The media were refreshed at 3-day intervals. After 21 days of osteogenic induction, the cells were fixed in 4% paraformaldehyde and then stained with Alizarin Red staining solution for 30 min at room temperature. To assess adipogenesis, cells were cultured in SD rat bone marrow mesenchymal stem cells adipogenic differentiation medium (Cyagen, China, RASMX-90031) following the manufacturer’s instructions. After 21 days of induction, cells were fixed in 4% paraformaldehyde for 30 min and were stained with Oil Red O for 30 min. The dishes were then washed twice with PBS and observed under a microscope [26].

### 2.4 CCK8 assay

CCK8 assay was performed to detect the growth curves of different concentrations of EMD on BMSCs. BMSCs was inoculated with 1000 cells per well in 96-well plates, cultured in the complete medium containing EMD (0, 5, 25, 50, 75, 100, 125 μg/ml, respectively) for 24 hours. After removing the original medium and adding the mixture containing 10 μl CCK8 (colleagues, Japan) + 100 μl medium (without serum) for 2 hours, the growth curve was drawn by measuring the absorbance value of (OD), by 450 nm [27].

### 2.5 Western blot analysis

BMSCs were seeded into 6-cm-diameter culture dishes and cultured in complete medium. EMD with a concentration of 5, 25, 50 μg/ml was added to the experimental group, respectively. And, the same amount of complete medium was added to the control group. The cells were collected after 3 days of culture. Total proteins were extracted from the BMSCs by ice-cold RIPA lysis buffer. Subsequently, lysate proteins were resolved by electrophoresis, followed by transfer to polyvinylidene difluoride (PVDF) membranes (Millipore, USA). Then, the membranes were blocked in 5% skim milk for 1 h and incubated overnight at 4℃ in diluted primary antibody. Finally, images of the target strip were developed in the way of using a Western Chemiluminescent HRP Substrate Kit (Millipore, USA). Primary antibodies directed against Runx2 (1:1500, Rabbit mAb#12,556, monoclonal antibody, Cell Signaling Technology, MA, USA), Osterix (1:1000, ab209484, monoclonal antibody, Abcam, UK), ALP (1:1500, ab5462, monoclonal antibody, Abcam, UK), β-catenin (1:6000, ab2572, Abcam, UK) and GAPDH (1:1000, bs-2188 R, Bioss, China). ImageJ was used to analyze the gray value of each band.

### 2.6 RNA extraction and real-time reverse transcription polymerase chain reaction (real-time RT-PCR) analysis

The total RNA is extracted using the AxyPrep reagent (Corning, New York, USA), and then the cDNA is synthesized in reverse direction according to manufacturer’s protocol (Takara Bio, Otsu, Japan). The cDNA was diluted 10 times, and mix with primers, double distilled water and PowerUp™ SYBR™ Green Master Mix (Applied Biosystems™, Thermo Fisher Scientific, USA). Finally, the quantitative polymerase chain reaction of the mixed solution was carried out on the Applied Biosystems ViiA 7 RT-PCR System. GAPDH was used as the internal reference gene, and the relative quantification was performed by the 2^−ΔΔCt^ method. Primers used were as follows: GAPDH, AAGTTCAACGGCACAGTCAAGG/ACGCCAGTAGACTCCACGACAT; Runx2, GAACCAAGAAGGCACAGACAGAA/GGCGGGACACCTACTCTCATACT; Osterix, AGTGGTATTGTAGGTGCTGTGGTC/AGTGGTATTGTAGGTGCTGTGGTC; ALP, AGTGGTATTGTAGGTGCTGTGGTC/AGTGGTATTGTAGGTGCTGTGGTC; OCN, AGTGGTATTGTAGGTGCTGTGGTC/AGTGGTATTGTAGGTGCTGTGGTC; COLI, AGAGGCATAAAGGGTCATCGTG/GAGAACCAGCAGAGCCAGGG.

### 2.7 Mineralization assay and osteogenesis of BMSCs on titanium surface

BMSCs were seeded at 4 × 10^4^ cells/well in 12-well plates, the experimental group was treated with 25 μg/ml EMD, while the control group was not treated with EMD. After 3 days, the medium was then replaced with osteogenic medium and the media was changed every 3 days. Calcium nodules were stained with alizarin red. Then, 10% sodium dodecyl sulfate was added to dissolve the calcium nodules. The absorbance value was measured at 405 nm with the Microplate Absorbance Reader.

BMSCs were inoculated on the surface of Sand-blasted Large grift Acid-etched titanium in 48-well plates, and the experimental group was added with 25 μg/ml EMD, while the control group was only added with the same amount of medium for continuous culture for 3 days, and then replaced with osteogenesis induction medium for culture. At the 8th week of osteogenesis induction, cells were fixed with 2% glutaraldehyde for 4 h, and the surface cells of titanium were dehydrated step by step, coated with gold-palladium, and scanned by electron microscope. Images were acquired from randomly selected areas of each specimen [28].

### 2.8 EMD activates Wnt/β-catenin signaling in BMSCs

BMSCs were inoculated on the surface of Sand-blasted Large grift Acid-etched titanium in 10-cm-diameter culture dishes, the control group was added with the same amount of medium (without serum), and the rest of the dishes were added with 25 μg/ml EMD, 10 μg/ml xav939 (Wnt signaling pathway inhibitor), 25 μg/ml EMD+ 10 μM xav939, respectively [29]. Total RNA and protein were extracted after 24 hours. The relative mRNA expressions of Runx2, Osterix, ALP, COLI and OCN were detected by qRT-PCR and the protein levels of ALP, Osterix, and Runx2 were detected by Western blot. Then, the same number of cells were inoculated on the surface of Sand-blasted Large grift Acid-etched titanium in 6-well plates, and the EMD concentrations of 0,5, 10, 15, 20, and 25 µg /ml were added into each well. After 3d culture, the expression of β-catenin and Osterix was detected by western blot.

Immunoprecipitation: BMSCs were lysed with lysis buffer. The supernatant fraction was incubated overnight with 3.5 μl β-catenin antibody (1:6000, ab2572, Abcam, UK) and 20 μl Protein A/G-Sepharose (GE Healthcare) at 4 ℃. Proteins were washed four times with PBS and detected by western blot [30].

### 2.9 Animal experiments

5*10^6^ BMSCs were seeded into 6-well dishes and incubated in complete medium. After cell attachment, 25 μg/ml EMD was added to the experimental group, and the same amount of medium was added to the control group. After 3 days, the plates were transferred into regular sheet-inducing medium (complete medium supplemented with 50 g/mL vitamin C). On day 14, the media were replaced with osteoinductive medium. On day 21 (after 7 days of osteogenic induction), the three-layer cell sheets were coated with titanium and then cultured in 5% CO_2_ incubator at 37℃ for 2 hours to promote the adhesion between the cell membrane and the material. Parallel incisions were made on both sides of the back of nude mice, and the implants were removed from the cell incubator and implanted into the back of nude mice. Each nude mouse was implanted into the control on the left back and the experimental group on the right side. In this experiment, 6-week-old male nude mice were selected, and all mice were fed in a specific-pathogen-free condition. The mice were sacrificed under CO_2_ anesthesia 4 weeks later, and the transplants were collected for histological analysis [31].

### 2.10 Statistical analysis

Our data are shown as the means ± standard error (SE). The experiments were commenced at least three times for the reproducibility. Statistical differences between two groups were determined in a way of one-way analysis of variance (ANOVA) or t test (and nonparametric tests), P value <0.05 (*) was deemed statistically significant.

## 3. Results

### 3.1 BMSCs were successfully isolated and cultured

**Figure 1.**
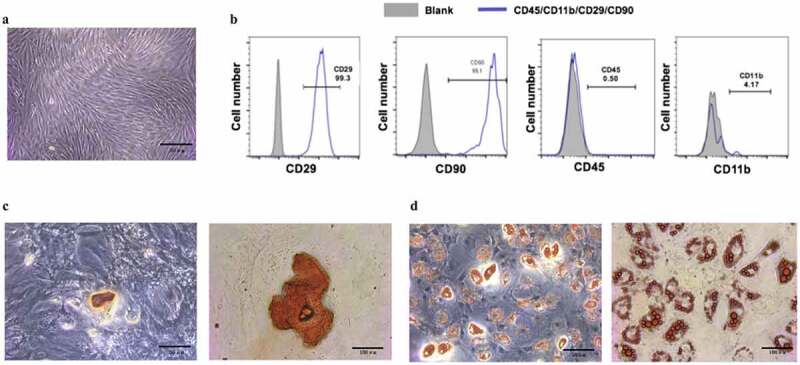
The culture of and the identification to bone marrow mesenchymal stem cells. (a) Bone marrow mesenchymal stem cells were cultured till their third generation. (b) Surface markers of BMSCs, CD45, CD90, were positive, while CD29,CD11b were negative. (c) Alizarin red staining was performed after four-week osteogenic differentiation. (d) Lipid droplets were observed by oil red O staining after 21d of adipogenic differentiation. (e) Isolated BMSCs colony was stained with crystal violet

In this study, we hypothesized that EMD could promote the proliferation and osteogenic differentiation of BMSCs through regulating Wnt/β-catenin signaling pathway. To investigate EMD’s effect on BMSCs and to analyze its mechanism, we firstly isolated the BMSCs. After 3–5 days of primary culture, the cells were spindle-shaped or shoal-shaped (Figure 1a). Flow cytometry showed that CD45, CD90, was positive, while CD29, CD11b was negative (Figure 1b). Four weeks after osteogenic differentiation, calcium nodules by alizarin red staining were positive, indicating that the cells had the ability of osteogenic differentiation (Figure 1c). Lipid droplets were observed 21 days after lipogenic differentiation (Figure 1d).

### 3.2 EMD promoted BMSCs proliferation

**Figure 2.**
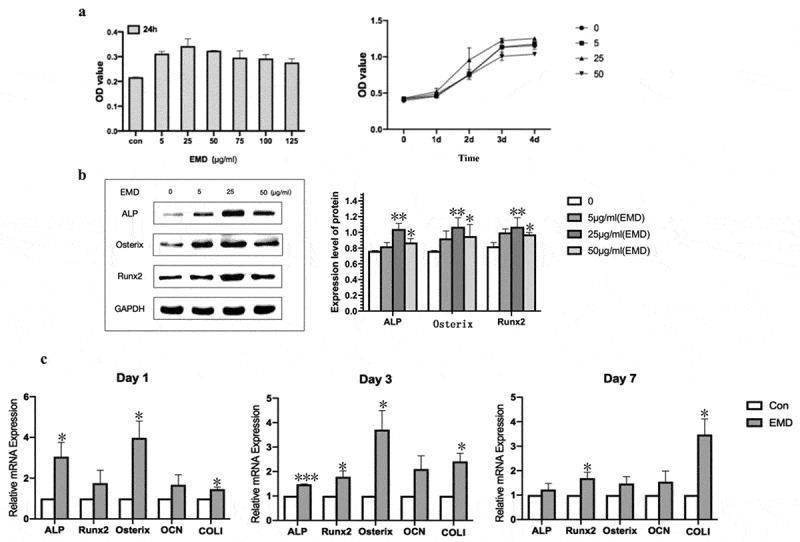
Optimal concentration of EMD and expression of mRNA. (a-b) The optimal concentration of EMD was 25 μg/ml. (c) The expression of osteogenesis-related genes was varying degrees higher when the concentration of EMD was 25 μg/ml. *P < 0.05, **P < 0.01 and ***P < 0.001 vs. the control

To detect the effect of EMD on the proliferation of BMSCs, CCK8 assay was performed. The results showed that different concentrations of EMD had different effects on the proliferation of stem cells, and the 24-h results showed that the effect of EMD at the concentration of 25 μg/ml and 50 μg/ml on the proliferation of stem cells was more significant than that of other concentrations (Figure 2a). The growth curve was further drawn, which showed that the OD value was the most significant after treated with EMD for 3 days.

### 3.3 EMD promoted the expression of osteoblastic differentiation markers

Western blot detected the protein levels of Runx2, Osterix, and ALP in cells treated with EMD of 5 μg/ml, 25 μg/ml, and 50 μg/ml for 24 h, respectively. The results showed that the expression levels of Runx2, Osterix, and ALP increased significantly at EMD concentration of 25 μg/ml compared with those of BMSCs treated with normal medium and EMD concentration of 5 μg/ml, 50 μg/ml (Figure 2b).

qRT-PCR was used to detect gene expression in cells on days 1, 3 and 7 (P3). The expression of related osteogenic genes (Runx2, Osterix, ALP, COLI) in BMSCs treated with 25 μg/ml EMD was to vary degrees higher than that of cells cultured in normal medium at the same time point (Figure 2c).

### 3.4 Effect of EMD on osteogenic differentiation of BMSCs

**Figure 3.**
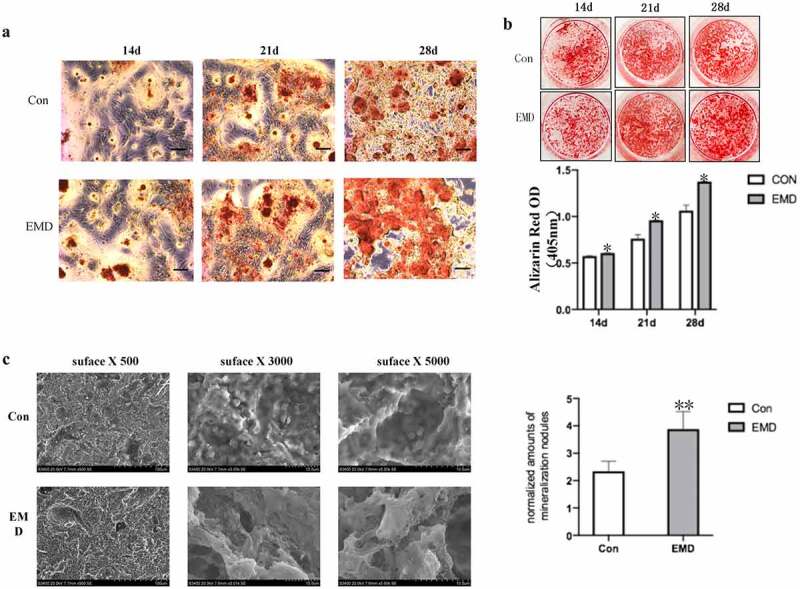
BMSCs’ cellular extracellular matrix mineralization subsequently the EMD stimulation. (a-b) The alizarin Red stained extracellular matrix mineralization of BMSCs after four-week EMD stimulation. The semi-quantitative measurments on the alizarin Red staining dye from the cultured cells within the period of BMSCs osteogenic differentiation after EMD stimulations was shown. (c) The number and the density of calcium nodules on the titanium surface were higher than those in the normal medium at the same time point. d. Protein level of Runx2 in BMSCs after three-day EMD treatment

Next, to detect the effect of EMD on the osteogenic differentiation of BMSCs, the osteogenic induction medium was added after treating with EMD for 3 days. At the 2nd, 3rd and 4th week of osteogenic differentiation induction, the number of calcification nodules stained with 0.2% alizarin red was higher than that of cells treated with normal medium at the same time point (Figure 3(a,b)). Semi-quantitative measurements of alizarin red showed similar results(Figure 3b).

Additionally, BMSCs were cultured and induced osteogenic differentiation on titanium surface, and EMD were added. After EMD treatment, at the 8th week of osteogenic induction differentiation, the number and density of calcium nodules on the titanium surface were higher than those in the normal medium at the same time point (Figure 3c). It is suggested that EMD can also enhance the potential of osteogenic differentiation of BMSCs on titanium surface.

### 3.5 Wnt/β-catenin signaling pathway was involved in regulating osteogenic differentiation

**Figure 4.**
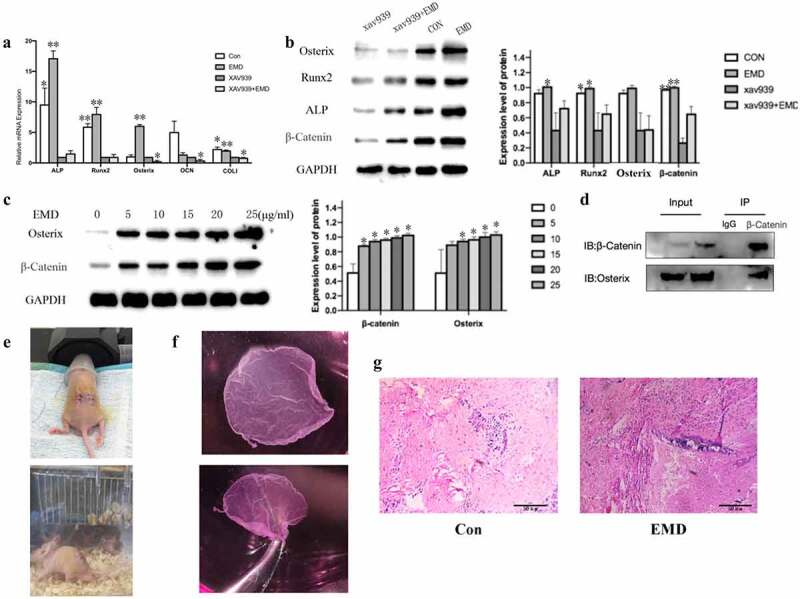
Wnt/β-catenin signaling’s relationship with EMD on bone marrow mesenchymal stem cells osteogenic differentiation and some vivo studies. (a-b) After adding inhibitors of Wnt classical pathway, the transcription factor β-catenin and the relative osteogenic gene expression decrease. *P < 0.05 and **P < 0.01 vs. the xav939 group. (c-d) Expression of Osterix was related with β – catenin. E-F 4 weeks after the cell sheets were implant in nude mice, there was not evident bone tissue formation in the control, but bone tissue formation was shown in the EMD treatment. *P < 0.05 and **P < 0.01 and ***P < 0.001 vs. 0 μg/ml EMD

After adding inhibitors (xav939) of Wnt classical pathway, qRT-PCR was performed to detected the mRNA expression of transcription factor β-catenin and the relative osteogenic gene expression of Runx2, Osterix, ALP, COLI, and OCN. The results showed that xav939 decreased the expression of Runx2, Osterix, ALP, COLI, and OCN (Figure 4a). Western blot results showed that the expression of β-catenin was also decreased when treated with xav939 (Figure 4b). And, the expression of β-catenin and its downstream protein Osterix increased with EMD concentration, in a concentration-dependent manner (Figure 4c). The results of co-immunoprecipitation indicated that the increased expression of Osterix was related to the increased expression of β-catenin (Figure 4d), which may be related to the activation of this signal pathway to regulate the process of osteogenic differentiation.

### 3.6 EMD promoted osteogenic differentiation in vivo

In vivo studies were performed to further verify the function of EMD on BMSCs osteogenic differentiation. The complex of BMSCs and titanium plate were given a subcutaneous transplantation on the back of nude mice (Figure 4(e,f)). After 4 weeks, HE staining results suggested that there was a small amount of red-stained suspected osteoid matrix and no massive bone formation in the control group, while a small amount of purple-blue immature bone tissue was observed in the experimental group (Figure 4g). The results showed that EMD promoted osteogenic differentiation in vivo.

## 4 Discussion

Stem cells’ discovery and the process of stem cell biology recently have made great contributions to regenerative treatment strategies for a variety of diseases [32]. Stem cells have two main properties: they can both renew themselves and differentiate. Compared with embryonic stem cells, usages of adult stem cells in clinical studies is less controversial because these cells can be harvested and differentiated without devastating the embryo [6]. Stem cell therapy with MSCs has obtained more and more popularity for bone diseases treatment over the years in clinical practices because of their high potential to differentiate into osteoblasts [33]. Additionally, MSCs are readily obtained from patients, cultivated ex vivo for clinical usages [34–36]. Previous studies in both vitro and vivo have shown that EMD prompts the regeneration of periodontal tissues and affects the proliferation and mineralization of both cementoblasts and periodontal ligament stem cells [18]. The results of this study suggest that the EMD efficacy on BMSCs proliferation is the most significant when the concentration of EMD is 25 μg/ml. Compared with using osteogenic induction medium alone to induce stem cells, BMSCs treated with EMD showed stronger mineralization ability.

Some studies have shown that EMD treatment can also make stem cells show more cell layers, secrete more extracellular matrix, and increase the expression of calcification-related genes and cementum-specific genes [37,38]. The follow-up qRT-PCR results of this experiment also confirmed that the expression of many important related genes, such as Runx2, Osterix, ALP, COLI, OCN, was up-regulated in varying degrees after adding EMD to the culture medium. Among them, ALP is known as an early marker of osteoblast differentiation, and transcription factors Runx2 and Osterix are necessary transcription factors for osteoblast differentiation and bone formation [39,40]. Runx2 is expressed at early stages of osteochondroprogenitor determination, followed by Osterix induction during osteoblast maturation [41]. In addition, Osterix manipulates many osteogenic factors expression, including osteonectin, osteopontin, osteocalcin, and ALP [42,43]. OCN is a matrix signal of bone formation and a specific marker for late osteogenic differentiation. It works as a regulator in the process of bone remodeling, and its expression level is usually consistent with the final mineralization level of matrix [44]. Type I collagen is the most abundant protein in bone content, accounting for more than 90% of bone mass, and provides tensile strength and support for bone connective tissue, so it is essential to evaluate its expression in the study of bone formation [44-45]. The results showed that the cells pretreated with EMD showed stronger osteogenic potential. The possible reason is that the proteins in EMD have a notable impact on cellular behavior in a way of mediating cell migration, diffusion, proliferation, and differentiation, as well as the expressions of signal molecules, growth factors, and extracellular matrix components.

Some in vitro studies have shown that visible protein aggregates were noted, when EMD was added to the media at physiological pH and room temperature [46]. Extracellular matrix mineralization is known as the basis of hard tissue construction, which includes two stages: the preliminary synthesis of collagen network and the deposition of hydroxyapatite crystal catalyzed via ALP [47]. In this study, in order to prove the relationship between EMD and osteogenic differentiation of BMSCs, we measured the mineralization ability of BMSCs before and after the treatment of EMD. Titanium is widely regarded as an ideal dental and bone implant because of its excellent biocompatibility and corrosion resistance. In this experiment, after sandblasting and acid etching on the surface of titanium implant, BMSCs cells were inoculated on titanium plate. The experimental group was pretreated with EMD and then osteogenic induction was performed. After 8 weeks of induction, the calcium nodules’ number in treatment group was greatly higher than that in the control, suggesting that EMD can also enhance the potential of BMSCs osteogenic differentiation on titanium surface. Semi-quantitative analysis of alizarin red also confirmed the above results, suggesting that EMD pretreated cells formed more calcium deposition, showing a stronger mineralization ability.

Wnt/β-catenin signaling pathway manipulates the growth and the development of many organs and tissues, from cell fate determination to differentiation, migration, and proliferation [48]. It is well known that β-catenin is an important regulator of the activation of Wnt pathway, which is essential for normal bone development, and the down-regulation of this pathway will damage bone formation [49]. In normal somatic cells, β-catenin merely functions as a cytoskeletal protein in cell membrane. E-cadherin formation complex takes part in maintaining the adhesion of homologous cells and preventing cell movement. Only when the extracellular Wnt signaling molecule binds to the specific receptor Frizzled protein on the cell membrane and activates the intracellular Disheveled protein, which leads to the inactivation of GSK3B and makes β-catenin avoid being phosphorylated and degraded, β-catenin can accumulate in the cytoplasm. When the concentration of β-catenin in the cytoplasm reaches a certain level, it can transfer to the nucleus. β-Catenin involves in this signaling pathway, and its accumulation in the cytoplasm, and then translocation into the nucleus is deemed as a sign of the activation of this signaling pathway. The Wnt/β-catenin pathway role in osteoblasts and bone marrow mesenchymal stem cells has been fully confirmed [50–52]. In this experiment, the expressions of osteogenesis-related genes and β-catenin were down-regulated after the addition xav939 and after EMD treatment, the mRNA and protein expressions of β-catenin and downstream Osterix increased with the increase of EMD concentration, which may be the accumulation of β-catenin in the cytoplasm, suggesting that the pathway was activated. Results of co-immunoprecipitation suggested that increasing level of Osterix expression was related to the increased expression of β-catenin, which may be related to the activation of this signal pathway to participate in the process of osteogenic differentiation, suggesting that EMD promoting BMSCs osteogenic differentiation might be associated with the participation of Wnt classical signal pathway. Does this process specifically regulate the role and process of other signal pathways? Further experimental study is needed.

Cell sheet-based tissue engineering is capable of establishing its unique traits and advantages for the usage in regenerative medicine and tissue modeling. Compared with a single cell, cell sheet is a better cell carrier, which enables it to provide its unique functions and advantages for regenerative medicine and tissue modeling [53]. Ascorbic acid is a simple method for cell sheet culture, which can obtain complete cell sheet by mechanical scraping after the cell sheet is mature. Compared to monolayer cell sheet, multilayer cell sheet has stronger mechanical properties. In this study, the cells were induced into cell sheet and then wrapped with titanium plate were implanted into nude mice to observe their osteogenesis. Nude mice lack immune response because they have no thymus. As recipients of heterotopic transplantation, rejection and inflammation caused by immune reaction can be avoided.

The results of HE staining suggested the possibility of bone formation of BMSC sheets on titanium surface in vivo. Compared with directly inducing BMSC to form diaphragm, the cell diaphragm formed by BMSC pretreated with EMD showed stronger potential of osteogenic differentiation after osteogenic induction and differentiation. Although the experiment in cell sheets and in vivo remains in its infancy, the outcomes might reveal that EMD is an important auxiliary tool to promote bone formation around the implant. It provides an option for subsequent experiments of bone tissue engineering.

## 5 Limitation and future direction

In this study, we focused on the role of the wnt-signaling pathway in the regulation of the osteogenic differentiation of BMSCs by EMD, and we have not yet explored the role of other signaling pathways or components in it. The microenvironment of organisms has complex components, and there are many regulatory factors in every life activity. In future research, we will continue to study other signaling pathways and the regulatory relationship between pathways.

## 6 Conclusions

We hence drew a conclusion that the EMD’s efficiency on BMSCs induced early and late osteogenic markers, which it’s a positive effect. Mechanistically speaking, we suggested that this process might be at least partially mediated through the Wnt/β-catenin pathway. Moreover, our exploration on the EMD effect in osteogenic differentiation of BMSCs provides a new idea for the treatment of bone deficiencies in the future. Although the idea of prefabricating EMD for BMSCs before osteogenic induction remains in its infancy, the outcomes from our study emphasize that it might prove to be an important tool for promoting bone formation nearby dental implants.

## Figures and Tables

**Table:** 

EMD:	enamel matrix derivative
BMSCs:	bone marrow mesenchymal stem cells
ALP:	alkaline phosphatase
OCN	osteocalcin
COL I:	collagen type I
Runx2	Runt-related transcription factor 2
PBS	phosphate-buffered saline; P/S: penicillin/streptomycin

## Data Availability

All data generated or analysed during this study are included in this published article

## Declarations

This research has been approved by the Animal Care Welfare

Committee of Guizhou Medical University.

## Highlights

EMD promoted the proliferation of BMSCs.

EMD promoted the osteogenic differentiation of BMSCs.

Wnt/β-catenin pathway was involved in EMD’s effect on osteogenic differentiation.

## References

[1] Tarafder S, Bose S. Polycaprolactone-coated 3D printed tricalcium phosphate scaffolds for bone tissue engineering: in vitro alendronate release behavior and local delivery effect on in vivo osteogenesis. ACS Appl Mater Interfaces. 2014;6(13):9955–9965. 24826838 10.1021/am501048n PMC4095936

[2] Avila-Ortiz G, Gubler M, Romero-Bustillos M, et al. Efficacy of alveolar ridge preservation: a randomized controlled trial. J Dent Res. 2020;99(4):402–409. 32050833 10.1177/0022034520905660

[3] Romanos GE, Delgado-Ruiz R, Sculean A. Concepts for prevention of complications in implant therapy. Periodontol. 2019;81(1):7–17. 10.1111/prd.12278 31407435

[4] Bose S, Sarkar N. Natural medicinal compounds in bone tissue engineering. Trends Biotechnol. 2020;38(4):404–417. 31882304 10.1016/j.tibtech.2019.11.005 PMC8015414

[5] Rauch A, Haakonsson AK, Madsen J, et al. Author correction: osteogenesis depends on commissioning of a network of stem cell transcription factors that act as repressors of adipogenesis. Nat Genet. 2019;51(4):766. 10.1038/s41588-019-0400-4 30911162

[6] Squillaro T, Peluso G, Galderisi U. Clinical trials with mesenchymal stem cells: an update. Cell Transplant. 2016;25(5):829–848. 26423725 10.3727/096368915X689622

[7] Miron RJ, Sculean A, Cochran DL, et al. Twenty years of enamel matrix derivative: the past, the present and the future. J Clin Periodontol. 2016;43(8):668–683. 26987551 10.1111/jcpe.12546

[8] Hama H, Azuma H, Seto H, et al. Inhibitory effect of enamel matrix derivative on osteoblastic differentiation of rat calvaria cells in culture. J Periodontal Res. 2008;43(2):179–185. 18302620 10.1111/j.1600-0765.2007.01010.x

[9] Margolis HC, Beniash E, Fowler CE. Role of macromolecular assembly of enamel matrix proteins in enamel formation. J Dent Res. 2006;85(9):775–793. 16931858 10.1177/154405910608500902

[10] Novaes AJ, Palioto DB. Experimental and clinical studies on plastic periodontal procedures. Periodontol. 2019;79(1):56–80. 10.1111/prd.12247 30892770

[11] Sculean A, Donos N, Windisch P, et al. Healing of human intrabony defects following treatment with enamel matrix proteins or guided tissue regeneration. J Periodontal Res. 1999;34(6):310–322. 10633886 10.1111/j.1600-0765.1999.tb02259.x

[12] Narukawa M, Suzuki N, Takayama T, et al. Enamel matrix derivative stimulates chondrogenic differentiation of ATDC5 cells. J Periodontal Res. 2007;42(2):131–137. 17305871 10.1111/j.1600-0765.2006.00926.x

[13] Ohyama M, Suzuki N, Yamaguchi Y, et al. Effect of enamel matrix derivative on the differentiation of C2C12 cells. J Periodontol. 2002;73(5):543–550. 12027258 10.1902/jop.2002.73.5.543

[14] Miron RJ, Oates CJ, Molenberg A, et al. The effect of enamel matrix proteins on the spreading, proliferation and differentiation of osteoblasts cultured on titanium surfaces. Biomaterials. 2010;31(3):449–460. 19819013 10.1016/j.biomaterials.2009.09.075

[15] Groeneveldt LC, Knuth C, Witte-Bouma J, et al. Enamel matrix derivative has no effect on the chondrogenic differentiation of mesenchymal stem cells. Front Bioeng Biotechnol. 2014;2:29. 25229057 10.3389/fbioe.2014.00029 PMC4151337

[16] Kemoun P, Gronthos S, Snead ML, et al. The role of cell surface markers and enamel matrix derivatives on human periodontal ligament mesenchymal progenitor responses in vitro. Biomaterials. 2011;32(30):7375–7388. 21784516 10.1016/j.biomaterials.2011.06.043 PMC4441221

[17] Wang Z, Feng Z, Wu G, et al. In vitro studies on human periodontal ligament stem cell sheets enhanced by enamel matrix derivative. Colloids Surf B Biointerfaces. 2016;141:102–111. 26844646 10.1016/j.colsurfb.2016.01.036

[18] Xia Y, Guo Y, Yang Z, et al. Iron oxide nanoparticle-calcium phosphate cement enhanced the osteogenic activities of stem cells through WNT/beta-catenin signaling. Mater Sci Eng C Mater Biol Appl. 2019;104:109955. 31500064 10.1016/j.msec.2019.109955

[19] Moorer MC, Riddle RC. Regulation of osteoblast metabolism by Wnt signaling. Endocrinol Metab (Seoul). 2018;33(3):318–330. 30112869 10.3803/EnM.2018.33.3.318 PMC6145954

[20] Jing D, Zhai M, Tong S, et al. Pulsed electromagnetic fields promote osteogenesis and osseointegration of porous titanium implants in bone defect repair through a Wnt/beta-catenin signaling-associated mechanism. Sci Rep. 2016;6(1):32045. 27555216 10.1038/srep32045 PMC4995433

[21] Osta B, Benedetti G, Miossec P. Classical and paradoxical effects of TNF-alpha on bone homeostasis. Front Immunol. 2014;5:48. 24592264 10.3389/fimmu.2014.00048 PMC3923157

[22] Gu Q, Chen C, Zhang Z, et al. Ginkgo biloba extract promotes osteogenic differentiation of human bone marrow mesenchymal stem cells in a pathway involving Wnt/beta-catenin signaling. Pharmacol Res. 2015;97:70–78. 25917209 10.1016/j.phrs.2015.04.004

[23] Long F. Building strong bones: molecular regulation of the osteoblast lineage. Nat Rev Mol Cell Biol. 2011;13(1):27–38. 22189423 10.1038/nrm3254

[24] Gao J, Xiang S, Wei X, et al. Icariin promotes the osteogenesis of bone marrow mesenchymal stem cells through regulating sclerostin and activating the Wnt/beta-Catenin signaling pathway. Biomed Res Int. 2021;2021:6666836. 33553429 10.1155/2021/6666836 PMC7847333

[25] Zhou J, Zhuang T, Ma P, et al. MicroRNA-547-5p-mediated interleukin-33/suppressor of tumorigenicity 2 signaling underlies the genesis and maintenance of neuropathic pain and is targeted by the therapy with bone marrow stromal cells. Mol Pain. 2020;16:2068231783. 10.1177/1744806920931737 PMC7309409 32513089

[26] Nemeth K, Wilson TM, Ren JJ, et al. Impaired function of bone marrow stromal cells in systemic mastocytosis. Stem Cell Res. 2015;15(1):42–53. 26001169 10.1016/j.scr.2015.04.005 PMC4557871

[27] Xu R, Zhang X, Xu Y, et al. Long noncoding RNA MST1P2 promotes cervical cancer progression by sponging with microRNA miR-133b. Bioengineered. 2021;12(1):1851–1860. 34034626 10.1080/21655979.2021.1921550 PMC8806230

[28] Yang HW, Lin MH, Xu YZ, et al. Osteogenesis of bone marrow mesenchymal stem cells on strontium-substituted nano-hydroxyapatite coated roughened titanium surfaces. Int J Clin Exp Med. 2015;8(1):257–264. 25784995 PMC4358450

[29] Deng Y, Lei G, Lin Z, et al. Engineering hyaline cartilage from mesenchymal stem cells with low hypertrophy potential via modulation of culture conditions and Wnt/beta-catenin pathway. Biomaterials. 2019;192:569–578. 30544046 10.1016/j.biomaterials.2018.11.036 PMC6733256

[30] Wang Z, Song K, Zhao W, et al. Dendritic cells in tumor microenvironment promoted the neuropathic pain via paracrine inflammatory and growth factors. Bioengineered. 2020;11(1):661–678. 32434423 10.1080/21655979.2020.1771068 PMC8291888

[31] Chen F, Feng X, Wu W, et al. Segmental bone tissue engineering by seeding osteoblast precursor cells into titanium mesh-coral composite scaffolds. Int J Oral Maxillofac Surg. 2007;36(9):822–827. 17804199 10.1016/j.ijom.2007.06.019

[32] Choi JR, Yong KW, Wan SW. Effect of hypoxia on human adipose-derived mesenchymal stem cells and its potential clinical applications. Cell Mol Life Sci. 2017;74(14):2587–2600. 28224204 10.1007/s00018-017-2484-2 PMC11107561

[33] Kristjansson B, Honsawek S. Current perspectives in mesenchymal stem cell therapies for osteoarthritis. Stem Cells Int. 2014;2014:194318. 25548573 10.1155/2014/194318 PMC4274908

[34] Elabd C, Centeno CJ, Schultz JR, et al. Intra-discal injection of autologous, hypoxic cultured bone marrow-derived mesenchymal stem cells in five patients with chronic lower back pain: a long-term safety and feasibility study. J Transl Med. 2016;14(1):253. 27585696 10.1186/s12967-016-1015-5 PMC5009698

[35] Keller CA, Gonwa TA, Hodge DO, et al. Feasibility, safety, and tolerance of mesenchymal stem cell therapy for obstructive chronic lung allograft dysfunction. Stem Cells Transl Med. 2018;7(2):161–167. 29322685 10.1002/sctm.17-0198 PMC5788872

[36] Cohen JA, Imrey PB, Planchon SM, et al. Pilot trial of intravenous autologous culture-expanded mesenchymal stem cell transplantation in multiple sclerosis. Mult Scler. 2018;24(4):501–511. 28381130 10.1177/1352458517703802 PMC5623598

[37] Wu SM, Chiu HC, Chin YT, et al. Effects of enamel matrix derivative on the proliferation and osteogenic differentiation of human gingival mesenchymal stem cells. Stem Cell Res Ther. 2014;5(2):52. 24739572 10.1186/scrt441 PMC4076631

[38] Hisanaga Y, Suzuki E, Aoki H, et al. Effect of the combined use of enamel matrix derivative and atelocollagen sponge scaffold on osteoblastic differentiation of mouse induced pluripotent stem cells in vitro. J Periodontal Res. 2018;53(2):240–249. 29044527 10.1111/jre.12511

[39] Artigas N, Urena C, Rodriguez-Carballo E, et al. Mitogen-activated protein kinase (MAPK)-regulated interactions between Osterix and Runx2 are critical for the transcriptional osteogenic program. J Biol Chem. 2014;289(39):27105–27117. 25122769 10.1074/jbc.M114.576793 PMC4175347

[40] Sinha KM, Zhou X. Genetic and molecular control of osterix in skeletal formation. J Cell Biochem. 2013;114(5):975–984. 23225263 10.1002/jcb.24439 PMC3725781

[41] Artigas N, Gamez B, Cubillos-Rojas M, et al. p53 inhibits SP7/Osterix activity in the transcriptional program of osteoblast differentiation. Cell Death Differ. 2017;24(12):2022–2031. 28777372 10.1038/cdd.2017.113 PMC5686339

[42] Nakashima K, Zhou X, Kunkel G, et al. The novel zinc finger-containing transcription factor osterix is required for osteoblast differentiation and bone formation. Cell. 2002;108(1):17–29. 11792318 10.1016/s0092-8674(01)00622-5

[43] Koga T, Matsui Y, Asagiri M, et al. NFAT and Osterix cooperatively regulate bone formation. Nat Med. 2005;11(8):880–885. 16041384 10.1038/nm1270

[44] Ahmed GM, Abouauf EA, Abubakr N, et al. Tissue engineering approaches for enamel, Dentin, and Pulp regeneration: an update. Stem Cells Int. 2020;2020:5734539. 32184832 10.1155/2020/5734539 PMC7060883

[45] Nguyen VT, Canciani B, Cirillo F, et al. Effect of chemically induced hypoxia on osteogenic and angiogenic differentiation of bone marrow mesenchymal stem cells and human umbilical vein endothelial cells in direct coculture. Cells-Basel. 2020;9:3. 10.3390/cells9030757 PMC7140659 32204578

[46] Apicella A, Heunemann P, Dejace L, et al. Scaffold requirements for periodontal regeneration with enamel matrix derivative proteins. Colloids Surf B Biointerfaces. 2017;156:221–226. 28531879 10.1016/j.colsurfb.2017.05.013

[47] Chen M, Huang L, Shen X, et al. Construction of multilayered molecular reservoirs on a titanium alloy implant for combinational drug delivery to promote osseointegration in osteoporotic conditions. Acta Biomater. 2020;105:304–318. 31982586 10.1016/j.actbio.2020.01.029

[48] Shi G, Yang F. Cion of human lens epithelial cells by enhancing the expression of zinc finger and BTB domain containing 7A (ZBTB7A) and activating Wnt/beta-catenin pathway. Bioengineered. 2021;12(1):4374–4384. 34304709 10.1080/21655979.2021.1953901 PMC8806501

[49] Zhong Z, Zylstra-Diegel CR, Schumacher CA, et al. Wntless functions in mature osteoblasts to regulate bone mass. Proc Natl Acad Sci U S A. 2012;109(33):E2197–E2204. 22745162 10.1073/pnas.1120407109 PMC3421196

[50] Feng X, Liu J, Xu Y, et al. Molecular mechanism underlying the difference in proliferation between placenta-derived and umbilical cord-derived mesenchymal stem cells. J Cell Physiol. 2020;235(10):6779–6793. 31990045 10.1002/jcp.29572

[51] Ling L, Nurcombe V, Cool SM. Wnt signaling controls the fate of mesenchymal stem cells. Gene. 2009;433(1–2):1–7. 19135507 10.1016/j.gene.2008.12.008

[52] Hay E, Dieudonne FX, Saidak Z, et al. N-cadherin/wnt interaction controls bone marrow mesenchymal cell fate and bone mass during aging. J Cell Physiol. 2014;229(11):1765–1775. 24664975 10.1002/jcp.24629

[53] Takahashi H, Okano T. Thermally-triggered fabrication of cell sheets for tissue engineering and regenerative medicine. Adv Drug Deliv Rev. 2019;138:276–292. 30639258 10.1016/j.addr.2019.01.004

